# Improvement of Restless Legs Syndrome Under Treatment of Cancer Pain With Morphine and Fentanyl

**DOI:** 10.3389/fneur.2019.00457

**Published:** 2019-05-08

**Authors:** Jan Gärtner, Karin Jaroslawski, Gerhild Becker, Christopher Boehlke

**Affiliations:** ^1^Palliative Care Center Hildegard, Basel, Switzerland; ^2^Faculty of Medicine, Medical Center, Clinic for Palliative Medicine, University of Freiburg, Freiburg, Germany

**Keywords:** restless-legs-syndrome, Willis-Ekbom disease, treatment, morphine, oxycodone, transdermal fentanyl

## Abstract

Restless-Legs-Syndrome (RLS), also known as Willis-Ekbom disease, is a sleep- and rest related disorder characterized by the unpleasant urge to move the legs. Pharmacological therapy is mainly based on dopamine-agonists and delta-2-alpha calcium channel ligands. Also, randomized-controlled-trials (RCTs) reported effectiveness of oral oxycodone (in combination with naloxone), and intrathecal opioids have also been administered for this indication. In the case reported here, a patient with advanced pancreatic cancer was referred to an acute palliative care unit for the treatment of cancer-related pain. Yet, in thorough exploration of her symptom burden, the patient reported that she felt her quality of life had been predominantly limited by symptoms other than cancer pain. Her medical history and neurological examination revealed that these symptoms were most obviously caused by severe RLS. In the years before, pharmacological therapies with dopamine-agonists and delta-2-alpha calcium channel ligands were initiated, but failed to relieve the RLS. In the palliative care ward, intravenous morphine was successfully titrated to treat her cancer pain. Concurrently, the patient also experienced almost complete relief from her RLS-symptoms and an increase in quality of life. The amelioration of her RLS-symptoms continued after morphine therapy was switched from intravenous to oral administration. Even after the patient was dismissed to home care and opioid rotation to transdermal fentanyl, symptom control of RLS remained excellent. To our knowledge, this is the first report of successfully treating RLS with intravenous and oral morphine. Since morphine is more easily available worldwide and the cost of morphine therapy is substantially lower compared to oxycodone/naloxone, comparisons to morphine may be an intriguing option for future RCTs.

## Introduction

Restless-Legs-Syndrome (RLS), also known as Willis-Ekbom disease, is a sleep- and rest related disorder characterized by unpleasant sensations in the legs and the urge to move the legs, which may reduce the symptoms (1). RLS is a common disease with a reported prevalence of 5–10% in European and North American adults, with 2–3% experiencing moderate-to-severe symptoms (2). Women are affected twice as frequently as men (2).

If non-pharmacological interventions fail to relieve symptom burden, common pharmacological interventions include dopamine-agonists or an alpha-2-delta calcium channel ligand as first-line therapy (3). Unfortunately, after initial amelioration of symptoms many patients (50–70% over a period of 10 years) treated with dopamine-agonists experience worsened symptoms under ongoing medication, a process called augmentation (4). Therefore, other therapy options are needed to help these patients.

As a second-line approach, opioids have been used in clinical practice for years, but only a few placebo-controlled randomized trials have been conducted to prove their effectiveness. A recent phase III trial showed reduction of RLS-symptoms when patients were treated with oxycodone/naloxone (5), but there are some concerns of attrition bias due to high drop-out rates (6). An older study investigated the monotherapy of oxycodone without naloxone showing similar beneficial effects on symptom burden (7).

Several retrospective studies implicate other opioids -for example methadone (4, 8) and tramadol (9)- to reduce RLS-symptoms. Additionally, intrathecal morphine was administered successfully to treat RLS-patients (10–12). However, to our knowledge so far there are no published reports on orally or intravenously administered morphine in the treatment of RLS.

## Case Report

A 75-year-old woman was admitted to our palliative ward with abdominal pain, nausea, and vomiting. The patient had been diagnosed with metastatic pancreas carcinoma with one singular liver metastasis 18 months before. She had received first- and second-line chemotherapy regimens; the latter had been stopped due to severe side effects. Two months prior to admission, when MRI scans revealed progressive disease, and together with her medical oncologist, the patient decided against continuing chemotherapy. Instead, symptom oriented, palliative care was chosen without any further antineoplastic therapy.

The patient had been suffering from RLS for 12 years already, with moderate to strong symptoms [Numerical Rating Scale (NRS): 6-10/10] mostly in the evening and at night. The family history regarding RLS was not investigated. She reported symptom alleviation by long walks (up to several hours long), and rigorous tennis playing, both of which she could no longer accomplish because of the progressive cancer related fatigue. Twelve years ago, her neurologist started treating RLS with levodpa, but after initial improvements in symptom control, symptoms began worsening again due to augmentation. Five years later, the patient was started on a transdermal application of the dopamine-agonist rotigotine (4 mg/d), but this treatment could not reduce RLS-symptoms satisfactorily. The patient reported that a trial of pregabalin was discontinued because of side effects (dizziness) and oxycodone was stopped because of nausea and vomiting. Thereafter transdermal rotigotine (4 mg/d) was continued with little effect until admission to our palliative care unit.

To assess RLS-symptom burden and pain we used the 11-NRS, an established tool to assess pain and commonly used in the palliative care setting, where 0 = no pain and 10 = worst possible pain (13). We used the NRS to semi-quantify RLS-symptom intensity, because it is well-known by staff while other assessment tools specifically designed for RLS are not established. When using the NRS for the assessment of RLS-symptoms, we asked the patient: how severe are your RLS-symptoms right now (0 = no RLS-symptoms, 10 = worst possible RLS-symptoms)?

Upon initial admission, she reported abdominal cramps (NRS 8/10). Her temperature and blood pressure were normal with a heart rate of 100 bpm. The abdomen was distended, but soft with normal bowel sounds. The patient reported ubiquitous abdominal tenderness. The rest of the physical examination was normal. Initial laboratory testing included elevated gamma-glutamyl transferase at 194 U/l (reference range, <40 U/L), lactate dehydrogenase at 364 U/L (reference range, 135–214 U/l) and C-reactive protein at 42 mg/l (reference range, <5 mg/l). Bilirubin and lipase levels were normal. The peripheral-blood count was normal. An abdominal ultrasound dismissed possible bowel obstruction, hepatic cholestasis, and gall bladder abnormalities, but revealed a significant amount of ascites, which is why percutaneous ascites drainage was performed (3.5l). Cell counts in the ascites fluid revealed elevated neutrophils/μl indicating spontaneous bacterial peritonitis. Calculated antibiotic treatment was started with tazobactam/piperacillin.

**Figure 1:**
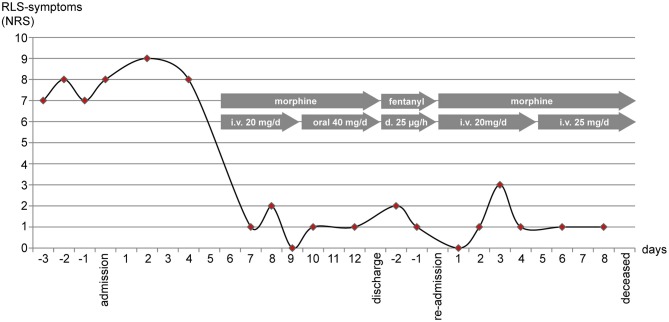
RLS-symptoms (the urge to move the legs) were measured using the numerating rating scale (NRS) once per day in the evening. After initiation of intravenous (i.v.) morphine (day 5) NRS-scores dropped. Scores remained low after switching morphine to oral administration, after rotation to transdermal (d.) fentanyl, and after continuing intravenous morphine when the patient was re-admitted to the hospital.

The patient also received intravenous fluids, analgesics (oral metamizole) and antiemetics (dimenhydrinate, ondansetrone). At day 5 after admission the abdominal pain exacerbated. Symptomatic analgesia with intravenous morphine (20 mg/d) was initiated. Pain management was excellent after 1 day with a NRS of 0-3/10. Unintendedly, the patient also reported almost complete symptom relief regarding her RLS (Figure 1), which had not occurred for her in years. After nausea and vomiting had resided, analgesics, including morphine were given orally. Still, pain management and the symptom control of RLS-symptoms remained steady. According to the patient's wish, she was discharged 13 days after admission. Three days later, she was re-admitted with increasing abdominal pain. Without our knowledge, her general practitioner had rotated morphine to transdermal fentanyl (25 μg/h). While pain control was insufficient, RLS-symptoms remained adequately controlled with this opioid therapy. After re-admission we discontinued transdermal fentanyl and re-initiated intravenous morphine therapy, which once again achieved excellent pain relief. Paracentesis revealed an increasing neutrophil count in the ascites. Considering her incurable, advancing and metastatic disease and good symptom control under analgesia, the patient declined antibiotic treatment and died a little more than 1 week later.

## Discussion

We report a case of a patient with advanced pancreatic cancer, treated with intravenous and oral morphine for cancer pain, who experienced markedly reduced symptom burden of her RLS-syndrome. While there are several publications reporting successful use of intrathecal morphine (10–12) and oral methadone and tramadol (4, 8, 9) in RLS, to our knowledge this is the first report showing amelioration of RLS-symptoms by morphine administered intravenously and orally and by transdermal fentanyl.

A systematic review by Trenkwalder et al. on association of RLS with certain diseases could not identify increased prevalence in malignant disease, although MEIS1, the RLS gene most strongly associated with RLS risk in GWAS studies, is a transcription factor with implications in leukemia and neuroblastoma (14). To our knowledge, no association with malignant disease has been reported so far. As in our patient, RLS symptoms occurred years before diagnosis of the malignant disease and no other typically RLS-associated condition was present. We therefore assume that she was suffering from primary (idiopathic) RLS. Still, a neoplastic origin from so far unidentified anti-neuronal antibodies (secondary RLS) cannot be excluded. The late onset of RLS in our patient might favor secondary disease, which is seen later in life than idiopathic disease (15).

The pathophysiology of RLS is poorly understood. Three main pathways seem to be involved: iron metabolism, dopaminergic dysfunction, and the central opioid system (16). Why are opioids effective in treatment of RLS? Cell culture experiments in iron-deficient conditions show that dopaminergic cells from the substantia nigra are protected from apoptosis by the delta-opioid peptide enkephalin (17). Furthermore, post mortem analyses of human brains showed reduced antibody staining against beta-endorphine and met-enkphalin in RLS patients when compared to controls, possibly involving the mu-opioid receptor subtype in the pathogenesis of RLS (18). At a morphological level, dendritic spines, which are small membranous protrusions at the dendrites proposed to be the cellular basis for learning and memory, may be involved in the pathogenesis of RLS (19, 20). Activation of ubiquitously clustered mu-opioid-receptors in excitatory synapses by morphine invoke morphological changes in dendritic spines and decreased expression of glutamate receptors (21). This may as well-contribute to the beneficial effect of opioids on RLS symptoms.

Our patient had previously been treated with the dopamine agonist rotigotine and levodopa. According to the practice guidelines, second line therapy should include delta-2-alpha calcium channel ligands such as gabapentin (level A recommendation) or pregabalin (level B) (22, 23). In our patient, pregabalin had caused dizziness and was discontinued. Some years before, her neurologist had treated our patient with oxycodone/naloxone (2 × 5 mg/2.5 mg/d; daily oral morphine equivalent dose of 15–20 mg), which had also not led to improvement of the RLS symptoms. Notably, the oxycodone/naloxone dose had not been increased stepwise, as it is suggested by the phase III trial (5). During her stay on our palliative care unit our patient was titrated up to 20 mg intravenous morphine daily dose (daily oral morphine equivalence of 40–60 mg), which is the equivalent dose of the fentanyl dose that relieved her RLS symptoms but around 200–300% the daily morphine equivalent when oxycodone/naloxone was tried unsuccessfully. This could explain why our patient had not experienced any benefits from oxycodone/naloxone concerning the RLS symptoms. We assume that opioid equivalents known from treatment of pain are also applicable to the treatment of RLS. This is not necessarily the case, because there might be other mechanisms involved during opioid-action in RLS. For example, several downstream targets of the mu-receptor are known (24). However, it is unknown, exactly which downstream targets are involved in mediation of mu-receptor activation in the treatment of RLS. These targets could be different in pain and RLS causing different equivalence dosages. In Europe, oxycodone/naloxone (Targin®) is approved for treatment of RLS after failure of dopaminergic therapies. No prospective RCTs have investigated the effectiveness of other opioids. Our patient experienced markedly reduced RLS-symptoms after initiation of analgesia with morphine, regardless of application route (intravenous or oral), and transdermal fentanyl. Interestingly, RLS symptom burden remained low after the patient's general practitioner switched oral morphine to transdermal fentanyl therapy. This indicates that in addition to oxycodone, morphine and other opioids may have beneficial effects on RLS-symptoms. RCTs with comparison of other strong opioids are warranted to investigate this intriguing option: morphine is more readily available worldwide and therapy costs of morphine are substantially lower compared to oxycodone/naloxone.

A concern in the long-term use of opioids is addiction. While opioids are well-established in treatment of cancer-pain, in chronic pain their use is controversial and should only be considered under certain precautions (25). There is no data investigating the issue of addiction when opioids are used for RLS. Therefore, opioid use in RLS-patients should be monitored closely to reduce potential abuse. Possible reversible causes of RLS-refractoriness (such as low iron stores) and other therapeutic options (such as pharmacological combination therapy, non-pharmacologic and complementary approaches) should be considered before prescribing opioids (26, 27). Although opioid use disorder could be a relevant problem in RLS-patients, we know that long-term use of opioids in “low” dosages (<100 mg/d morphine or equivalent) has significantly lower risks than the use of high dosages (26, 28). Additionally, a recent study found increased rates of invasive pneumococcal pneumonia in patients receiving opioid therapy (29). In the cohort study of Wiese et al. the authors hypothesize that this finding may be caused by the immunosuppressant effects of opioids, but confounders and other risks of bias cannot be excluded. Nevertheless, these findings and the debate about the current “opioid epidemic” emphasize the need for thorough risk-benefit appraisal for each individual patient before initiating opioid therapy for RLS (30).

## Concluding Remarks

A patient with advanced pancreatic cancer experienced pronounced and sustained amelioration of RLS-symptoms by intravenous and oral morphine therapy and due to transdermal fentanyl therapy. In the literature no case reports or studies of oral or intravenous morphine or transdermal fentanyl against RLS could be identified. As morphine is more readily available worldwide and therapy costs of morphine are substantially lower compared to oxycodone/naloxone, randomized clinical trials are warranted to investigate the role of morphine in the treatment of RLS. Yet, in the non-palliative care population, thorough individual risk-benefit appraisal should be conducted for every patient before initiating opioid therapy due to safety issues concerning misuse (addiction) and potential immunosuppression.

## Ethics Statement

The patient provided verbal and written consent for use of the patient's personal health information in this case report.

## Author Contributions

CB and JG wrote the manuscript. CB prepared the figure, and GB and KJ edited the final manuscript.

### Conflict of Interest Statement

JG has received research grants, speaker's fees and honoraria for advisory boards from several manufacturers of opioids. These include: Grünenthal, Mundipharma, Teva, and Sanofi-Aventis. The remaining authors declare that the research was conducted in the absence of any commercial or financial relationships that could be construed as a potential conflict of interest.
